# Intra-Organ Variation in Age-Related Mutation Accumulation in the Mouse

**DOI:** 10.1371/journal.pone.0000876

**Published:** 2007-09-12

**Authors:** Rita A. Busuttil, Ana Maria Garcia, Robert L. Reddick, Martijn E. T. Dollé, Robert B. Calder, James F. Nelson, Jan Vijg

**Affiliations:** 1 Buck Institute for Age Research, Novato, California, United States of America; 2 University of Texas at San Antonio, San Antonio, Texas, United States of America; 3 University of Texas Health Science Center, San Antonio, Texas, United States of America; 4 National Institute of Public Health and The Environment, Bilthoven, The Netherlands; Baylor College of Medicine, United States of America

## Abstract

Using a transgenic mouse model harboring chromosomally integrated *lacZ* mutational target genes, we previously demonstrated that mutations accumulate with age much more rapidly in the small intestine than in the brain. Here it is shown that in the small intestine point mutations preferentially accumulate in epithelial cells of the mucosa scraped off the underlying serosa. The mucosal cells are the differentiated villus cells that have undergone multiple cell divisions. A smaller age-related increase, also involving genome rearrangements, was observed in the serosa, which consists mainly of the remaining crypts and non-dividing smooth muscle cells. In the brain we observed an accumulation of only point mutations in no other areas than hypothalamus and hippocampus. To directly test for cell division as the determining factor in the generation of point mutations we compared mutation induction between mitotically active and quiescent embryonic fibroblasts from the same *lacZ* mice, treated with either UV (a point mutagen) or hydrogen peroxide (a clastogen). The results indicate that while point mutations are highly replication-dependent, genome rearrangements are as easily induced in non-dividing cells as in mitotically active ones. This strongly suggests that the point mutations found to have accumulated in the mucosal part of the small intestine are the consequence of replication errors. The same is likely true for point mutations accumulating in hippocampus and hypothalamus of the brain since neurogenesis in these two areas continues throughout life. The observed intra-organ variation in mutation susceptibility as well as the variation in replication dependency of different types of mutations indicates the need to not only extend observations made on whole organs to their sub-structures but also take the type of mutations and mitotic activity of the cells into consideration. This should help elucidating the impact of genome instability and its consequences on aging and disease.

## Introduction

Genomic instability is one of the hallmarks of cancer and has also been implicated in other aging-related diseases as well as in the process of aging itself [1]. Although there is abundant evidence for mutation accumulation in cells and tissues during aging, the functional significance of increased genomic instability for the various age-related degenerative processes is as yet unclear. Indeed, most work on mutagenesis has been done using *in vitro* systems, and the relevance of these findings for the situation in mammalian organs and tissues during aging is not always clear.

Using transgenic mice harboring a chromosomally integrated *lacZ* reporter gene our laboratory has previously demonstrated an age- and tissue-specific increase in spontaneous mutations *in vivo* [2,3]. Interestingly, the small intestine, a tissue with a very high cell proliferative activity, was found to undergo the largest age-related increase in mutant frequency among all organs studied, i.e., from 11.0×10^−5^ in young mice to 25.6×10^−5^ in old mice [3]. By contrast, in brain, a postmitotic organ, there was virtually no such age-related increase, i.e., from 4.8×10^−5^ in young animals to 5.0×10^−5^ in old ones [2]. It is conceivable that the high rate of mutation accumulation in small intestine is due to replication errors, which are less likely to occur in the brain, a mainly postmitotic organ. To investigate if this interpretation is correct we decided to comparatively analyze *lacZ* mutant frequencies in potentially relevant substructures of these two organ systems.

For the brain we selected the hypothalamus and hippocampus, together with the cortex, as potentially important functional targets of spontaneous mutagenesis. The hippocampus is known to be critical for the formation of new memory and highly vulnerable to damage, as evident from its association with neurodegenerative disease [4]. Likewise, the hypothalamus is a small but critical part of the brain, producing a number of peptide releasing factors that control a host of vital needs that depend on hormonal balance [5]. The cortex plays a role in many complex brain functions such as memory and awareness. For the small intestine a comparison between the inner, mucosal layer of epithelial cells of the villi, with the presumably largest number of completed cell divisions, and the outer cell layer, the serosa, with a much lower number of completed cell divisions was chosen as the most relevant one.

The results generally confirm a relation between increased mutagenesis and high cell proliferative activity, but this proved to be dependent on the type of mutation. While point mutations were found to have accumulated more rapidly in the mucosal layer of the intestine, which mainly consists of epithelial cells that had undergone a number of cell divisions, genome rearrangement mutations also accumulated in the serosa, which after scraping off the mucosa mainly consists of remaining crypts and smooth muscle cells with much less completed rounds of cell division. Direct comparison of mutation induction between mitotically active and quiescent embryonic fibroblasts indicated that only point mutations and not the larger genome rearrangements are replication-dependent. Indeed, in the brain, we observed high levels of point mutations in the hippocampus and hypothalamus, which were found to increase with age in contrast to the situation in the brain overall. These mutations could therefore be caused by replication errors during neurogenesis, which is known to continue in these areas throughout life.

## Materials and Methods

### Transgenic animals

A cohort of C57/Bl6 pUR288-lacZ mice of line 30 (integration site on chromosome 11) were maintained in the animal facilities of the University of Texas Health Science Center at San Antonio. The mice were maintained on a 14-hour light/10-hour dark cycle at a standard temperature of 23°C. Standard lab chow (Harlan Teklad, USA) and water were supplied *ad libitum*. Animals were sacrificed by cervical dislocation following CO_2_ inhalation.

### Organ removal and dissection of sub-parts

Immediately after sacrificing the animals the total brains were carefully removed and placed in a petri dish on ice. Then, the hypothalamus, hippocampus and the cortex were excised, frozen on dry ice and separately kept at −80°C together with the remainder of the total brain until later use. The small intestine was removed, rinsed in PBS and its contents emptied. After opening it by slicing lengthwise, the mucosal surface was gently washed in PBS, after which the intestine was separated into duodenum, jejunum and ileum. Of each intestinal subpart the mucosal layer, histopathologically identified by characteristic evagination into plicae and villi, was scraped off under a dissection microscope. The mucosa and serosa of each animal were frozen at −80°C until later use.

### Generation of proliferating and quiescent cells

Mouse embryonic fibroblasts (MEFs) were obtained from individual embryos derived as described [6]. Proliferating and quiescent cell populations were prepared as described earlier [7]. Briefly, proliferating cell populations were generated by seeding early passage MEFs in 10-cm dishes (1,000,000 cells). Cells were cultured in 10% serum and the medium was replaced every 3–4 days for the duration of the experiment. Proliferating cells were treated 24 hours after seeding. To generate quiescent populations, cells were seeded as described for proliferating cells. After 24 h, the cells were washed 3 times with PBS and given DMEM containing 0.5% serum. The cells were maintained in this medium throughout the period of the experiment and used after 5 days. For UV irradiation, proliferating or quiescent cells were washed twice with PBS, covered with a thin layer of PBS and irradiated in lidless culture dishes using a germicidal lamp (254 nm, 15 W, General Electric, USA). Cells were then returned to culture. For proliferating cells, fresh medium was provided. For quiescent cells, the low-serum medium, which had been retained, was returned to the plates. For H_2_O_2_ treatment proliferating or quiescent cells were washed twice with PBS and then incubated with 0.1 mM H_2_O_2_ (Sigma-Aldrich, USA) in medium without serum for 2 h at 37°C. After the treatment period the cells were provided with either fresh or the old medium as after UV irradiation and further cultured. Control cells were mock treated. Cells were harvested 72 hours after treatment.

### [^3^H]-thymidine incorporation

[^3^H]-thymidine incorporation was measured as described previously [7]. Briefly, six hours prior to harvesting, 1 µCi[^3^H]-TdR was added to each well of six-well plates containing proliferating or quiescent cell populations and incubation was continued at 37°C for 6 h. Cells were then washed twice with ice-cold PBS and once with ice-cold 5% trichloroacetic acid (TCA). After the addition of 1 ml TCA, the cells were placed at 4°C for 30 min, washed twice with PBS, and solubilized with 500 µl 0.5 M NaOH, 0.5% SDS. The solubilized cells were mixed with 10 ml scintillation cocktail plus 100 µl glacial acetic acid, and radioactivity was quantified using a scintillation counter (Beckman Instruments, USA).

### Plasmid rescue and mutant frequency determination

DNA was extracted by routine phenol/chloroform extractions. Complete protocols for plasmid rescue and mutant frequency determinations with this model have been described elsewhere [8]. Briefly, between 20 and 30 µg genomic DNA was digested with *Hind* III (Roche, Switzerland) for one hour in the presence of magnetic beads (Invitrogen, USA) pre-coated with *lacZ*-*lacI* fusion protein. The beads were washed three times to remove excess genomic DNA. Plasmids were subsequently eluted from the beads by IPTG. After circularization of the plasmids with T4 DNA ligase they were ethanol precipitated and used to electrotransform *Escherichia coli C* (▵*lacZ*, *galE* ^-^) cells. One thousandth of the transformed cells was plated on the titer plate containing X-gal and the remainder on the selective plate containing p-gal. The plates were incubated for 15 h at 37°C. Mutant frequencies were determined as the number of colonies on the selective plate divided by the number of colonies on the titer plate (times the dilution factor of 1,000). Each mutant frequency is based on at least 300,000 recovered plasmids.

### Mutant classification and characterization

Mutant colonies were taken from the selective plates and grown at 37°C overnight in 96-well round-bottomed plates containing LB medium, kanamycin and ampicillin. One µl was then directly plated on X-gal to screen for galactose insensitive host cells and their background was subtracted [9]. One µl was added to a PCR mix and the DNA amplified as described previously [6]. The PCR products were digested with *Ava*I and size separated on a 1% agarose gel. Mutant plasmids with restriction patterns resembling the wild-type pattern were classed as “no-change” mutations whilst those deviating from the wild-type restriction pattern were classified as “size-change” mutations. Approximately 48 mutants per time point were analyzed. *LacZ* genes of selected mutant plasmids were prepared for sequencing. Sequence reactions of purified mutant plasmids were outsourced to Davis Sequencing (Davis, USA). The returned chromatograms were analyzed with Sequencher (Gene Codes, USA). The primers used for the sequence reactions were the same as described earlier [9].

### Statistical analysis

Statistical analysis was carried out using R [10]. The mean cumulative mutant frequencies obtained for the brain and small intestine were log transformed. An analysis of variance model was fit on the transformed mutant frequencies [11]. Corrected p-values were calculated using Tukey's Honest Significant Difference method for tissue, age and tissue:age [12]. T-tests were used for all other analyses. All tests were analyzed at a 0.05 level of significance.

## Results

### Mutation accumulation in brain subparts

**Figure 1:**
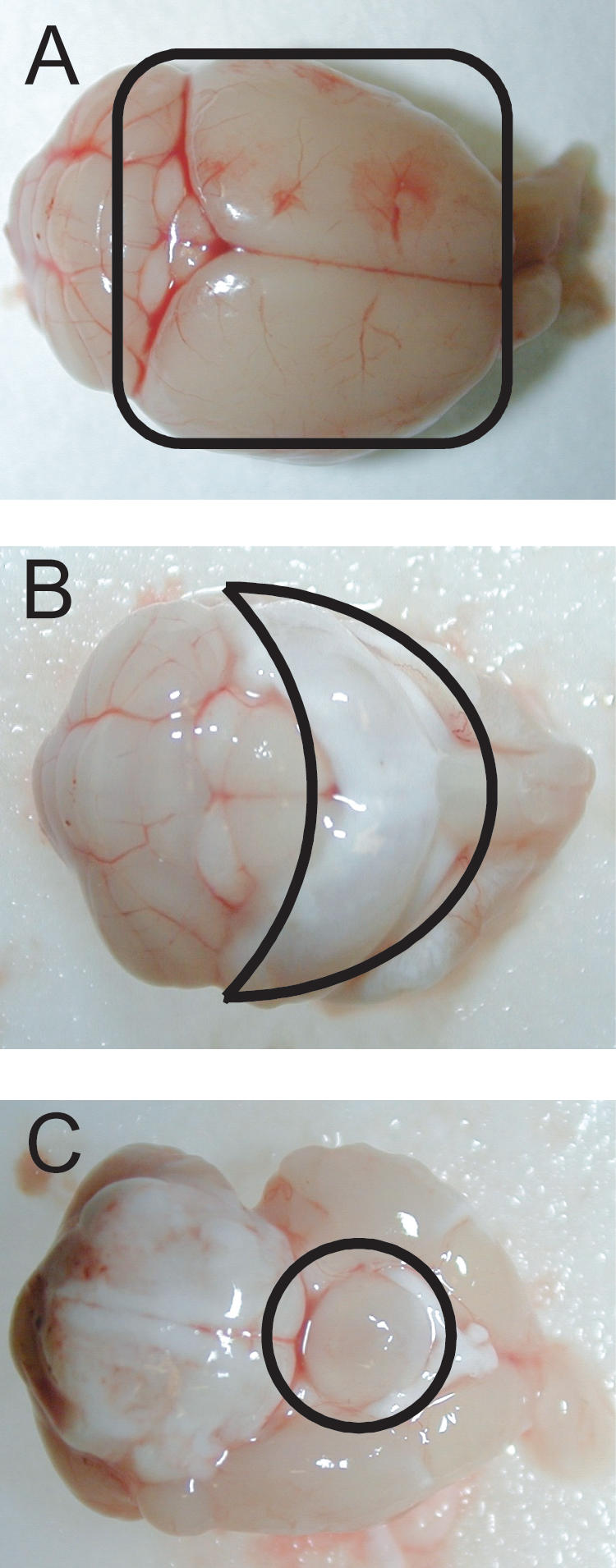
Division of the mouse brain was divided into 4 subparts (A) cortex; (B) hippocampus; (C) hypothalamus. The remainder is not shown.

**Figure 2:**
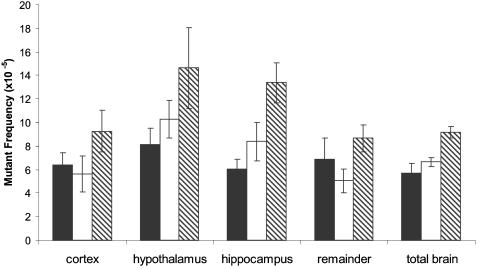
Spontaneous *lacZ* mutant frequencies in brain subparts and total brain. 7-month (black bars), 19-month (white bars) and 30-month (hatched) old mice.

Figure 1 shows the hippocampus, hypothalamus and cortex as they were removed from the mouse brain. DNA was extracted and mutant frequencies determined for each of these subparts as well as for the remaining part of the brain at 7-, 19-and 30-months of age, and compared with that of the total brain at these age levels. For each time point 3–6 animals were analyzed. The results (Figure 2) indicate that hippocampus and hypothalamus are the regions of the brain with the highest mutant frequency. Mutant frequencies in these sub-parts also increased significantly with age. The mean mutant frequency in hippocampus at 7 months was 6.1×10^−5^ and increased to 13.4×10^−5^ at 30 months of age (p<0.00001). Similarly, at 7 months of age, the hypothalamus showed a mean mutant frequency of 8.1×10^−5^ increasing to 14.6×10^−5^ in 30-month old animals (p = 0.0032). While previous results already indicated a slight age-related increase in mutant frequency for the total brain [2], in this experiment such an increase was also statistically significant, i.e., from 5.7×10^−5^ at 7 months to 9.2×10^−5^ at 30 months of age (p = 0.0318). It is possible that this increase is entirely due to the increase in mutant frequency of hippocampus and hypothalamus. Indeed, neither in cortex nor in the remainder of the brain was a statistically significant increase in *lacZ* mutant frequency observed (although there was a tendency for the mutant frequency at the oldest age to be higher than at younger ages). Combined across age, a significant difference was observed when the mean mutant frequency of hippocampus or hypothalamus were compared to the mean mutant frequency of cortex (p = 0.0305; p<0.00001), remainder (p = 0.0148; p<0.0001) or when the mutant frequency of hypothalamus was compared to total brain (p<0.0001) (Table S1). The overall mean mutant frequency of hypothalamus was also slightly but significantly higher than the mean mutant frequency of hippocampus (p = 0.0267). The results presented here indicate that there is significant intra-organ variation in spontaneous mutant frequency accumulation in the brain, with hippocampus and hypothalamus most susceptible.

**Figure 3:**
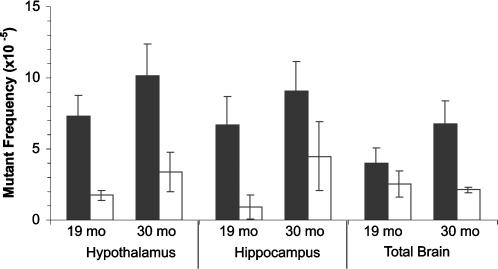
Frequency of *lacZ* point mutants or genome rearrangements in hypothalamus, hippocampus or total brain of 19-month and 30-month old mice. Point mutants: black bars; genome rearrangements: white bars.

In order to gain further insight into the nature of the excess mutations found in the hypothalamus and hippocampus we characterized mutants from the 19-and 30-month old brain subparts at the molecular level. For mutant characterization, the entire *lacZ* gene was PCR amplified and subjected to restriction digestion to distinguish between no-change mutants (those that exhibited the wild-type restriction pattern and consist of single-base changes as well as small insertions and deletions up to 50 bp) and size-change mutants (those that deviate from the wild-type restriction pattern and mostly represent rearrangements with one breakpoint in the *lacZ* gene and the other elsewhere in the mouse genome). As shown in Figure 3, most of the mutations observed in the hippocampus and hypothalamus were of the no-change class, in both age groups.

**Figure 4:**
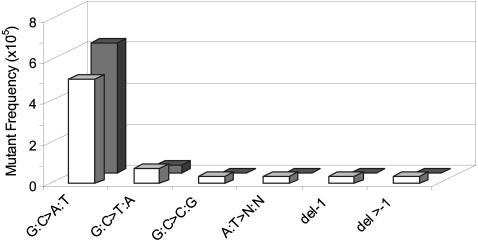
Point mutational spectra of hypothalamus and hippocampus of 19-month old mice. Hypothalamus: white bars; hippocampus: gray bars. Bars indicate the frequency of each type of point mutation as indicated.

To further investigate the nature of the no-change mutations in the hypothalamus and hippocampus, about 20 no-change mutants isolated from five, 19-month-old animals were randomly selected and subjected to sequencing. The mutational spectra did not deviate significantly between the two tissues and mainly consisted of GC to AT transition mutations (Figure 4).

### Mutant frequency determination in the small intestine

**Figure 5:**
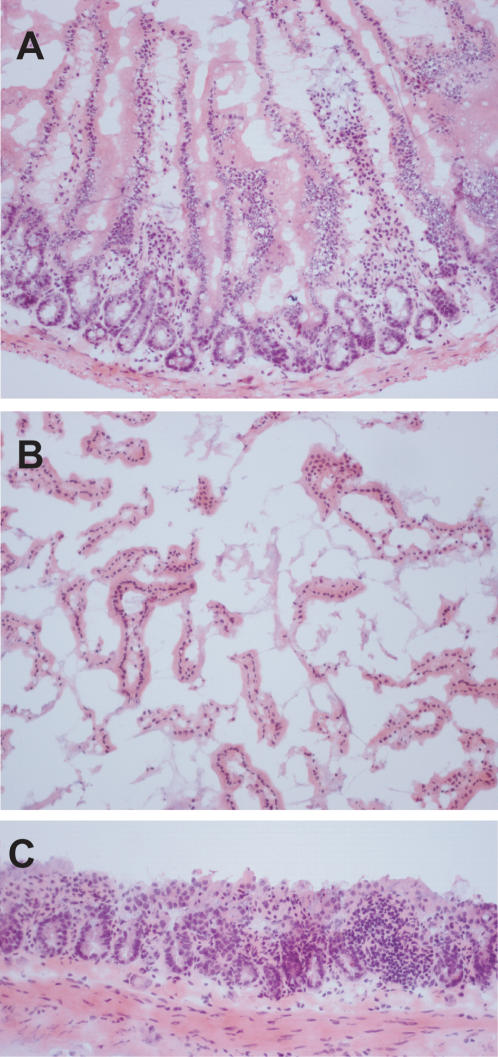
Sub dissection of the small intestine. (A) Intact mucosa prior to scraping. The surface epithelium is of normal configuration and the villi prominent (original magnification: 10×). (B) Mucosa, scraped off the serosa. This consisted mainly of the portion of the villi superficial to the crypts. No muscle was present in the specimen (original magnification: 20×). (C) The serosa after scraping. The surface villi were removed and the crypts and muscularis appeared to be partially intact (original magnification: 20×).

**Figure 6:**
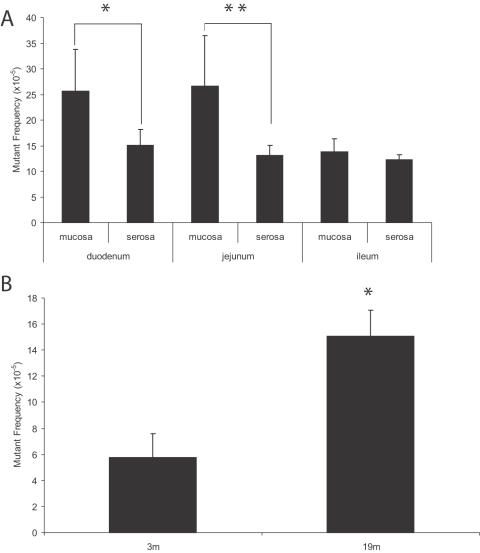
Mutant frequencies in intestinal subparts. (A) Spontaneous *lacZ* mutant frequencies in the mucosal or serosal layers of the duodenum, jejunum or ileum of 19-month old mice. Significant differences were observed between the inner (mucosa) layer and the outer (serosa) layer in both the duodenum (*; p = 0.0047) and the jejunum (**; p = 0.0003) regions of the small intestine. (B) Total *lacZ* mutant frequencies in the serosal layer of the duodenum of 3-month and 19-month old mice (p<0.0001).

While the time course of mutant frequencies in the sub-parts of the brain was impossible to predict, for the small intestine the question was merely whether the observed age-related increase in mutant frequency resided mainly in the inner layer of villi, containing the differentiated epithelial cells that are the result of multiple rounds of cell division during their differentiation. This proved to be the case. To capture most of the epithelial cells, we scraped off the innermost layer of the intestine (mucosa) with the surface villi. Figure 5A shows the intact mucosa. We then determined the *LacZ* mutant frequencies in the mucosal prep (Figure 5B) and in the remaining fraction, which consists of the serosa, muscularis and part of the lamina propia with some crypts still visible (Figure 5C). For brevity we call this the serosa throughout the text. These two fractions were analyzed for the duodenum, jejunum and ileum from 3 to 6 mice per determination point at 19 months (Figure 6A, Table S2). At this age most of the mutations accumulated over the life time of the animal are already present [3]. The mean mutant frequencies in the mucosal layer of duodenum (25.6×10^−5^) and jejunum (25.7×10^−5^) were significantly higher than those observed in the serosal layer of duodenum (15.1×10^−5^; p = 0.0047) or jejunum (13.1×10^−5^; p = 0.0003). In contrast, ileum did not show a statistically significant difference in mutant frequency between mucosa and serosa layers. However, mutations were also found to accumulate with age in the serosa. This was evident from a direct comparison of the total *lacZ* mutant frequency in serosa of the duodenum between young (3-mo) and old (19-mo) animals (Figure 6B; p<0.0001).

**Figure 7:**
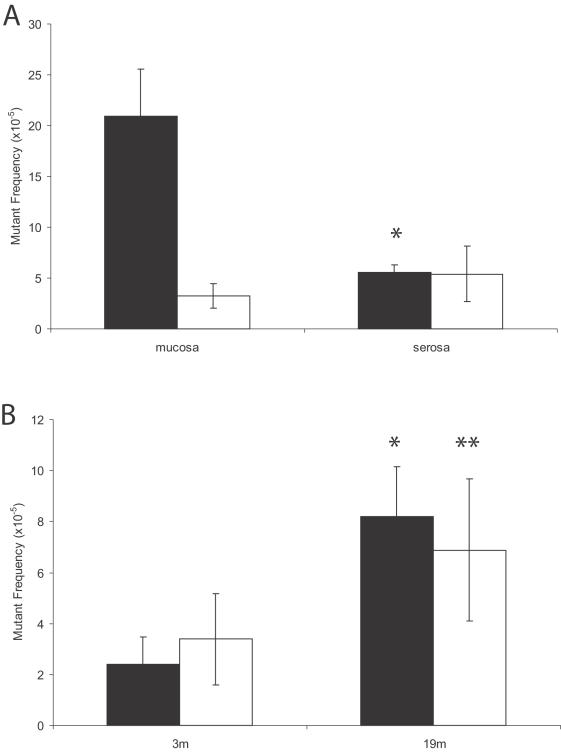
Mutant spectra in intestinal subparts. (A) Mean *lacZ* mutant frequencies of point mutants (black bars) and genome rearrangements (white bars) in the mucosal and serosal sublayers of the duodenum derived from 19-month old mice. There was a significant (*; p = 0.0003) difference in the frequency of point mutants between the sublayers. (B) Mutant spectra in the duodenum of young (3 m) and old (19 m) mice. The frequency of both point mutations (black bars) and genome rearrangements (white bars) mutants was found to increase with age (*; p = 0.0004 and **; p = 0.0459, respectively).

**Figure 8:**
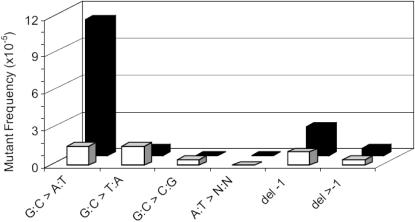
Point mutational spectra in the mucosal and serosal sublayers of duodenum from 19-month old mice. Mucosal layer: black bars; serosal layers: white bars.

We further investigated the molecular nature of the mutations in the duodenum of 19-month old mice, which together with the jejunum displayed a significant difference in mutant frequency between mucosa and serosa. Using the same restriction analysis strategy as described above for the brain subparts, we observed that the duodenum mucosal layer is highly susceptible to no-change mutation accumulation (Figure 7A) with only a small fraction of the mutations falling in the size-change class. By contrast, no-change mutations and size changes occurred in the serosal layer of the duodenum at equal frequencies (Figure 7A). Indeed, both these types of mutations were found to accumulate, modestly but significantly, in the serosa between 3 and 19 months (Figure 7B). We cannot exclude the possibility that the accumulated point mutations in the serosa are really derived from parts of the mucosa which were not entirely removed from the serosa (Figure 5C). Sequence analysis of 20 mutant plasmids indicated that the difference in point mutational spectra between the two layers at 19 months of age is entirely due to the increased level of GC to AT transitions in the mucosa. Point mutations in the serosa layer displayed equal fractions of GC to AT transitions and GC to TA transversions with some 1-base pair deletions (Figure 8). All mutations were different, indicating a lack of a founder effect in the form of jackpot mutations. In view of the modest standard deviations in mutant frequencies between animals jackpot mutations were not expected.

### Replication independent mutation accumulation

**Figure 9:**
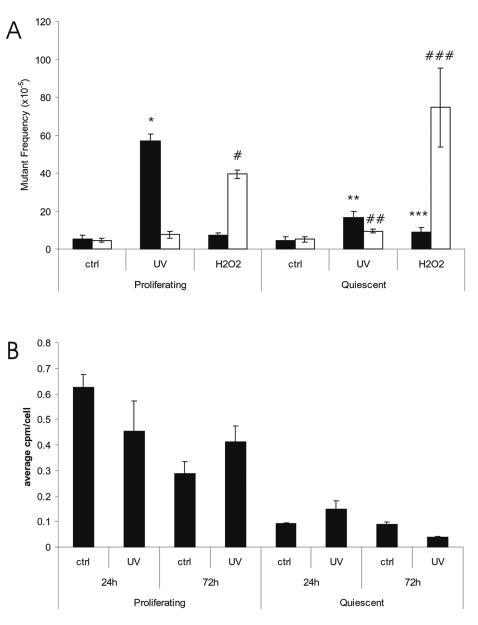
Mutant frequencies in proliferating and quiescent mouse embryonic fibroblasts. (A) Frequencies of point mutations (black bars) and genome rearrangements (white bars) in *lacZ*-MEFs, either irradiated with 2.5 J/m^2^ UVC radiation or treated with 0.1 mM H_2_O_2_ at 3-days post treatment. Mutant frequencies of treated cells were compared to their relevant control. A significant increase in no-change mutations was found in proliferating cells following UV exposure (*; p = 0.0002) whilst in cells treated with H_2_O_2_ only a significant increase in the frequency of size-change mutations was observed (#, p = 0.0234). In the quiescent cells a significant increase in both no-change (**; p = 0.0012) and size-change (##; p = 0.0119) mutations was observed following UV treatment. Similarly both no-change (***; p = 0.0302) and size-change (###; p = 0.0297) mutation frequencies increased after H_2_O_2 _exposure. (B) [^3^H]-thymidine incorporation in proliferating and quiescent cells harvested 24-and 72 h after treatment with 2.5 J/m^2^ UVC radiation. Background levels of thymidine incorporation were subtracted. The Y-axis represents the average counts per cell in the population tested.

Based on the results thus far obtained we hypothesized that only point mutations, but not genome rearrangements are strictly dependent on replication errors in mitotically active cell populations. Indeed, only point mutations were observed to have accumulated in the epithelial mucosa while the considerably smaller number of mutations accumulated in the serosa were, at least in part, genome rearrangements. Also the point mutations accumulating in hippocampus and hypothalamus could be explained by replication errors in these areas of the brain which are known to be sites of neurogenesis. To investigate whether mutations can be induced in non-replicating cell populations, we treated embryonic fibroblasts (MEFs) derived from the *lacZ*-plasmid transgenic mice with two different DNA damaging agents, 2.5 J/m^2^ UV or 0.1 mM H_2_O_2_, to induce damage, and then analyzed the mutant frequency in proliferating and quiescent cell populations. Cell survival at these doses is more than 80% (not shown). *LacZ* mutant frequency determinations in these cell populations indicated that while UV-induced mutagenesis is highly dependent on cell proliferation, H_2_O_2_ treatment resulted in slightly more mutations in the quiescent cells than in the actively proliferating ones (Figure 9A; previously published in preliminary form as conference proceedings [13]). Interestingly, while the replication-dependent, UV-induced mutations were mainly point mutations, H_2_O_2_ treatment of the MEFs resulted almost exclusively in the kind of genome rearrangement mutations (Figure 9A) observed to increase with age in the serosa of the small intestine (Figure 7B) and in heart and liver of these same *lacZ*-plasmid mice [2], [3]. Of note, the UV dose applied was small enough to allow rapid recovery of DNA synthesis after an initial growth arrest. This was demonstrated by measuring the incorporation of [^3^H]-thymidine both at 24 and 72 h after UV exposure (Figure 9B), but was also evident from cell counts (not shown). Hence, these results suggest that point mutations are highly dependent on replication errors, while genome rearrangements are not. This latter type of mutation might be a result of errors during annealing of DNA double-strand breaks known to be induced by H_2_O_2_. Such events do require DNA replication for its fixation.

## Discussion

We have previously reported an age-related accumulation of mutations in the mouse that is highly organ-specific [2], [3]. The contrast between the large increase of mutations in the small intestine and the virtual absence of such an increase in brain was especially striking. Based on the dramatic difference between these two organ systems with respect to their mitotic activity [14] it is reasonable to ascribe the rapid mutation accumulation in the small intestine to replication errors. In this present work we demonstrate that replication errors associated with high mitotic activity are indeed the most likely explanation of the rapid age-related mutation accumulation in the mouse small intestine. Our present results clearly indicate a significantly higher frequency of point mutations in the inner layer of the small intestine- the site containing cells that should have undergone the most rounds of cell divisions- than the outer part, containing mostly smooth muscle cells and some remaining crypts. This conclusion of a major role of replication errors in the generation of spontaneous mutations *in vivo* is further strengthened by our present finding of an increased mutation load in those parts of the brain, i.e., hippocampus and hypothalamus, that are known centers of neurogenesis. Also in this case the excess mutations were all point mutations. What could be the cause of the age-related increase in DNA mutations in these tissue substructures and what are the possible consequences?

In the small intestine we previously reported diverse types of point mutations accumulating during aging. It is likely that they are a consequence of error prone replication through a DNA template damaged by a variety of chemical lesions that find their origin in normal dietary components. However, in part, spontaneous mutations in the small intestine could also derive from erroneous processing of oxidative DNA damage, for example, as by-products of metabolism of food components. While metabolic activation occurs mainly in liver, there is evidence that also the intestine is subject to DNA damage induced by free radical intermediates of detoxified food components [15]. Interestingly, Ono et al. [16] also analyzed mutation accumulation in the mouse small intestine and also report a steep age-related increase of point mutations in the epithelial component. They ascribed the mutation accumulation to oxidative stress, possibly due to bile acids, which have been reported to induce the generation of reactive oxygen species [17]. Interestingly, bile from the liver and secretions from the pancreas come through the ampulla of Vater to mix with food in the duodenum. In the duodenum and the jejunum, but not in the more distal ileum, we see the significantly higher mutation frequency in mucosa as compared to the serosa.

In the hippocampus and hypothalamus virtually the only type of point mutation observed was the GC to AT transition, a likely consequence of oxidative damage [18]. A significant age-dependent increase in the generation of free radicals and antioxidant defenses in the critical hypothalamic-pituitary-adrenal (HPA) axis, i.e., including hippocampus and hypothalamus, has been reported by Siqueira et al. [19]. It is possible that the observed increased mutation frequencies that we observed in these same sub-structures of the mouse during aging are solely due to increased oxidative stress. It is possible that at least in part, this increased level of genome instability is causally related to the well-documented cognitive impairment and dysregulation of the HPA axis, with the hippocampus and hypothalamus both implicated in this phenomenon [19], [20].

Apart from point mutations, the *lacZ* reporter system is also capable of detecting genome rearrangements, i.e., events such as deletions and translocations that result from the erroneous reannealing of double-strand breaks in a *lacZ* reporter gene and elsewhere in the genome, either on the same chromosome or on another chromosome. Our present results indicate that such mutations increase with age in the serosal part of the small intestine. Using UV radiation to induce mutations at the *lacZ* locus in proliferating mouse embryonic fibroblasts we have previously shown that point mutations are strongly dependent on mitotic activity [7]. Our present finding that in quiescent MEFs the well-known clastogenic agent H_2_O_2_ induces at least the same amount of genome rearrangement mutations as in mitotically active cells indicates a crucial difference between the two categories of mutagenesis. Apparently, genome rearrangement mutations, possibly induced as a consequence of erroneous endjoining, require no cell division and they can therefore easily accumulate in postmitotic cells. This also explains our previous observation that such mutations accumulate in the heart during aging [3].

Interestingly, the observed replication-independent nature of genome rearrangements is in keeping with the very similar frequency of such mutations in the male and female germ line as a cause of heritable disease [21]. This is in contrast to disease-causing point mutations, which occur much more frequently in males than in females, with the highest incidence occurring in older males [22]. Since sperm are continuously produced throughout reproductive life, the number of cell divisions that have occurred increases with age and hence so does the total number of mutations. The number of cell divisions that are required to produce a human sperm cell is much greater than the number required to produce an egg cell. The ovum of a human female undergoes only 22 cell divisions prior to meiosis [23], which may explain the relative lack of increase in point mutations with age in females as compared to males. While it is not possible to generalize because of differences in both species and cell type, it is intriguing that the association of germ line point mutations with increased cell division is in complete agreement with our results of point mutations in somatic cells being highly dependent on cell proliferation whilst rearrangements can occur in both dividing and non-dividing cell populations.

As yet we have no explanation as to why in the brain only point mutations accumulate against a background of a very low level of genome rearrangement mutations that does not change with age. This is in contrast with the heart, another postmitotic organ, in which large genome rearrangements are very frequent and accumulate with age. It is possible that the brain, as the most highly complex organ in terms of the number of genes expressed [24], has a low tolerance for genome rearrangements, which are likely to distort patterns of long-range gene regulation [25]. Alternative possibilities are that the sources of spontaneous DNA damage differ from organ to organ and/or the DNA repair systems that are active in removing the lesions.

Mutation accumulation has been implicated as a causal factor in aging and its associated disease phenotypes, most notably cancer. Loeb and co-workers have stressed the role of reactive oxygen species in the pathogenesis of common tumors based on certain signature mutations, i.e., C to T transitions and C to A transversions [26]. In this present work we show that such mutations do arise spontaneously *in vivo*, most likely as a consequence of errors in replicating DNA damaged by reactive oxygen species. However, we also show that age-related mutation accumulation can depend on cell type and organ sub-structure, and that not all mutations are a consequence of replication errors. These results underscore the need to carefully define both the cell type in which spontaneous mutations arise *in vivo* and their molecular nature.

## Supporting Information

Table S1 (0.12 MB DOC)

Table S2 (0.06 MB DOC)
