# Supplementary material for: Intra-Organ Variation in Age-Related Mutation Accumulation in the Mouse
Source: PLoS One. 2007 Sep 12;2(9):e876. doi: 10.1371/journal.pone.0000876 (PMC1964533; doi:10.1371/journal.pone.0000876)
Supplement: Table S1 — (0.12 MB DOC) [file pone.0000876.s001.doc]

**Table S1.** Spontaneous mutant frequencies in the intestinal tract subparts of 7-, 19- and 30-month old animals.

| **Condition** |  |  | **1** | **2** | **3** | **4** | **5** | **6** |
| --- | --- | --- | --- | --- | --- | --- | --- | --- |
| **Cortex 7 mo** | No. of colonies |  | 901 | 722 | 1200 | 818 | 689 | 1073 |
|  | No. of mutants |  | 64 | 41 | 59 | 64 | 42 | 72 |
|  | MF (x10^-5^) |  | 7.10 | 5.68 | 4.92 | 7.82 | 6.10 | 6.71 |
|  | Mean MF (x10^-5^) | 6.39 ± 1.04 |  |  |  |  |  |  |
| **Remainder 7 mo** | No. of colonies |  | 1456 | 799 | 1043 | 927 | 1124 | 1209 |
|  | No. of mutants |  | 106 | 42 | 62 | 52 | 113 | 88 |
|  | MF (x10^-5^) |  | 7.28 | 5.26 | 5.94 | 5.61 | 10.05 | 7.28 |
|  | Mean MF (x10^-5^) | 6.90 ± 1.76 |  |  |  |  |  |  |
| **Hypothalamus 7mo** | No. of colonies |  | 683 | 891 | 574 | 503 | 628 | 778 |
|  | No. of mutants |  | 52 | 60 | 48 | 41 | 45 | 83 |
|  | MF (x10^-5^) |  | 7.61 | 6.73 | 8.36 | 8.15 | 7.17 | 10.67 |
|  | Mean MF (x10^-5^) | 8.12 ± 1.39 |  |  |  |  |  |  |
| **Hippocampus 7 mo** | No. of colonies |  | 434 | 951 | 713 | 536 | 492 | 822 |
|  | No. of mutants |  | 30 | 59 | 44 | 25 | 28 | 56 |
|  | MF (x10^-5^) |  | 6.91 | 6.20 | 6.17 | 4.66 | 5.69 | 6.81 |
|  | Mean MF (x10^-5^) | 6.08 ± 0.83 |  |  |  |  |  |  |
| **Total brain 7 mo** | No. of colonies |  | 2474 | 2641 | 2451 | 1286 | 2940 | 1797 |
|  | No. of mutants |  | 160 | 132 | 154 | 57 | 181 | 106 |
|  | MF (x10^-5^) |  | 6.47 | 5.00 | 6.28 | 4.43 | 6.16 | 5.9 |
|  | Mean MF (x10^-5^) | 5.71 ± 0.81 |  |  |  |  |  |  |
| **Cortex 19 mo** | No. of colonies |  | 968 | 1177 | 1020 | 826 | 654 |  |
|  | No. of mutants |  | 53 | 92 | 53 | 29 | 40 |  |
|  | MF (x10^-5^) |  | 5.48 | 7.82 | 5.20 | 3.51 | 6.12 |  |
|  | Mean MF (x10^-5^) | 5.62 ± 1.56 |  |  |  |  |  |  |
| **Remainder 19 mo** | No. of colonies |  | 791 | 1774 | 1797 | 1516 | 1834 |  |
|  | No. of mutants |  | 44 | 73 | 83 | 99 | 80 |  |
|  | MF (x10^-5^) |  | 5.56 | 4.11 | 4.62 | 6.53 | 4.36 |  |
|  | Mean MF (x10^-5^) | 5.04 ± 1.00 |  |  |  |  |  |  |
| **Hypothalamus 19 mo** | No. of colonies |  | 750 | 1016 | 837 | 748 | 912 |  |
|  | No. of mutants |  | 58 | 102 | 95 | 77 | 108 |  |
|  | MF (x10^-5^) |  | 7.73 | 10.04 | 11.35 | 10.29 | 11.84 |  |
|  | Mean MF (x10^-5^) | 10.25 ± 1.59 |  |  |  |  |  |  |
| **Hippocampus 19 mo** | No. of colonies |  | 635 | 589 | 325 | 488 | 923 |  |
|  | No. of mutants |  | 49 | 54 | 35 | 32 | 71 |  |
|  | MF (x10^-5^) |  | 7.72 | 9.17 | 10.77 | 6.56 | 7.69 |  |
|  | Mean MF (x10^-5^) | 8.38 ± 1.63 |  |  |  |  |  |  |
| **Total brain 19 mo** | No. of colonies |  | 4556 | 4176 | 2083 |  |  |  |
|  | No. of mutants |  | 295 | 296 | 132 |  |  |  |
|  | MF (x10^-5^) |  | 6.47 | 7.09 | 6.34 |  |  |  |
|  | Mean MF (x10^-5^) | 6.63 ± 0.40 |  |  |  |  |  |  |
| **Cortex 30 mo** | No. of colonies |  | 801 | 357 | 521 |  |  |  |
|  | No. of mutants |  | 61 | 32 | 58 |  |  |  |
|  | MF (x10^-5^) |  | 7.62 | 8.96 | 11.13 |  |  |  |
|  | Mean MF (x10^-5^) | 9.24 ± 1.77 |  |  |  |  |  |  |
| **Remainder 30 mo** | No. of colonies |  | 984 | 640 | 657 |  |  |  |
|  | No. of mutants |  | 73 | 62 | 58 |  |  |  |
|  | MF (x10^-5^) |  | 7.42 | 9.69 | 8.83 |  |  |  |
|  | Mean MF (x10^-5^) | 8.64 ± 1.15 |  |  |  |  |  |  |
| **Hypothalamus 30 mo** | No. of colonies |  | 661 | 744 | 893 |  |  |  |
|  | No. of mutants |  | 74 | 109 | 161 |  |  |  |
|  | MF (x10^-5^) |  | 11.20 | 14.65 | 18.03 |  |  |  |
|  | Mean MF (x10^-5^) | 14.62 ± 3.42 |  |  |  |  |  |  |
| **Hippocampus 19 mo** |  |  | 1356 | 1190 | 607 |  |  |  |
|  |  |  | 155 | 167 | 89 |  |  |  |
|  |  |  | 11.43 | 14.03 | 14.66 |  |  |  |
|  |  | 13.38 ± 1.71 |  |  |  |  |  |  |
| **Total Brain 30 mo** | No. of colonies |  | 3548 | 3085 | 2731 |  |  |  |
|  | No. of mutants |  | 343 | 267 | 251 |  |  |  |
|  | MF (x10^-5^) |  | 9.67 | 8.65 | 9.19 |  |  |  |
|  | Mean MF (x10^-5^) | 9.17 ± 0.51 |  |  |  |  |  |  |
