# Supplementary material for: Intra-Organ Variation in Age-Related Mutation Accumulation in the Mouse
Source: PLoS One. 2007 Sep 12;2(9):e876. doi: 10.1371/journal.pone.0000876 (PMC1964533; doi:10.1371/journal.pone.0000876)
Supplement: Table S2 — (0.06 MB DOC) [file pone.0000876.s002.doc]

| **Condition** |  |  | **1** | **2** | **3** | **4** | **5** | **6** |
| --- | --- | --- | --- | --- | --- | --- | --- | --- |
| **Duodenum** | No. of colonies |  | 3700 | 3198 | 3403 | 2240 | 1302 | 3048 |
| **mucosa** | No. of mutants |  | 996 | 828 | 666 | 448 | 534 | 610 |
|  | MF (x10^-5^) |  | 26.92 | 25.89 | 19.57 | 20.00 | 41.01 | 20.01 |
|  | Mean MF (x10^-5^) | 25.57 ± 8.23 |  |  |  |  |  |  |
| **Duodenum** | No. of colonies |  | 5560 | 4400 | 4968 | 2150 | 1760 | 2439 |
| **serosa** | No. of mutants |  | 793 | 836 | 814 | 352 | 170 | 360 |
|  | MF (x10^-5^) |  | 14.26 | 19.00 | 16.38 | 16.37 | 9.66 | 14.76 |
|  | Mean MF (x10^-5^) | 15.07 ± 3.13 |  |  |  |  |  |  |
| **Jejunum** | No. of colonies |  | 2176 | 3568 | 2544 | 1277 | 2160 | 2698 |
| **mucosa** | No. of mutants |  | 416 | 761 | 545 | 552 | 601 | 569 |
|  | MF (x10^-5^) |  | 19.12 | 21.33 | 21.42 | 43.23 | 27.82 | 21.09 |
|  | Mean MF (x10^-5^) | 25.67 ± 9.10 |  |  |  |  |  |  |
| **Jejunum** | No. of colonies |  | 5576 | 4376 | 6356 | 2968 | 2434 | 3484 |
| **serosa** | No. of mutants |  | 652 | 725 | 879 | 379 | 261 | 448 |
|  | MF (x10^-5^) |  | 11.69 | 16.57 | 13.83 | 12.77 | 10.72 | 12.86 |
|  | Mean MF (x10^-5^) | 13.07 ± 2.02 |  |  |  |  |  |  |
| **Ileum** | No. of colonies |  | 2836 | 3252 | 5420 | 4652 | 2832 | 3232 |
| **mucosa** | No. of mutants |  | 351 | 472 | 903 | 562 | 467 | 341 |
|  | MF (x10^-5^) |  | 12.38 | 14.51 | 16.66 | 12.08 | 16.49 | 10.55 |
|  | Mean MF (x10^-5^) | 13.78 ± 2.51 |  |  |  |  |  |  |
| **Ileum** | No. of colonies |  | 4760 | 4820 | 4822 | 1757 | 3706 | 2274 |
| **serosa** | No. of mutants |  | 559 | 648 | 598 | 201 | 413 | 300 |
|  | MF (x10^-5^) |  | 11.74 | 13.44 | 12.40 | 11.44 | 11.14 | 13.19 |
|  | Mean MF (x10^-5^) | 12.23 ± 0.95 |  |  |  |  |  |  |

**Table S2.** Spontaneous mutant frequencies in the intestinal tract subparts of 19-month old animals.
